# Functional Outcomes of Bipolar Hemiarthroplasty in Unstable Intertrochanteric Femur Fractures in the Elderly: A Prospective Study

**DOI:** 10.7759/cureus.65731

**Published:** 2024-07-30

**Authors:** Aalekh Rathod, Devashis Barick, Akhilesh S Khobragade, Virendra E Patil, Suhas Waghe, Aniruddha A Vaidya

**Affiliations:** 1 Orthopaedics and Trauma, Grant Government Medical College and Sir J.J. Group of Hospitals, Mumbai, IND; 2 Orthopaedics and Traumatology, N. K. P. Salve Institute of Medical Sciences & Research Centre and Lata Mangeshkar Hospital, Nagpur, IND; 3 Orthopaedics, N. K. P. Salve Institute of Medical Sciences & Research Centre and Lata Mangeshkar Hospital, Nagpur, IND

**Keywords:** merle d'aubigné score, proximal femoral nail, harris hip score, functional outcome, intertrochanteric femur fracture, bipolar hemiarthroplasty

## Abstract

Background

Intertrochanteric femur fractures are relatively common injuries among elderly individuals. Treatment options include fixation of intertrochanteric fractures using proximal femoral nails (PFNs), dynamic hip screws (DHSs), and unipolar and bipolar hemiarthroplasty. Unstable types of intertrochanteric fractures (Arbeitsgemeinschaft für Osteosynthesefragen (AO) types 31-A2 and A3) are more common in elderly osteoporotic people. Intertrochanteric femur fractures having a subtrochanteric extension, comminution at the posteromedial cortex, and reverse obliquity are considered to be unstable. The purpose of the study is to evaluate the functional outcomes of an unstable intertrochanteric femur fracture treated with bipolar hemiarthroplasty as the primary treatment option.

Aims and objectives

This study aims to assess the functional outcomes of bipolar hemiarthroplasty in unstable intertrochanteric fractures in the elderly using the Harris Hip Score (HHS) and the Merle d'Aubigné and Postel Criteria. The time point of assessment was from August 2016 to August 2018.

Material and methods

Fifteen elderly patients with unstable intertrochanteric fractures of the femur were treated with cemented bipolar hemiarthroplasty. Patients with unstable femur fractures or with osteoporosis and age above 65 years were included in the study. Harris Hip Score and the Merle d'Aubigné and Postel Criteria were used to measure functional outcomes. Patients were followed up at the first, third, and sixth months and subsequently at the end of one year.

Results

The mean age of the patients was 78.73 years. The majority (11) of the patients were female (73%). The average operative time was 119 minutes, the average blood loss was 380 ml, the mean postoperative hospital stay was 12 days, and the average time taken for mobilization was four days. An average of 15 elderly patients with unstable intertrochanteric fractures of the femur were treated with cemented bipolar hemiarthroplasty. The HHS on the first follow-up was 42.44 (SD of 6.52), followed by a score of 64.43 (SD of 8.11) on the second follow-up, 82.32 (SD of 2.81) on the third follow-up, and 84.23 (SD of 3.15) on the fourth follow-up. Eleven patients had good results, and two had fair results at the one-year follow-up, according to the HHS. According to the Merle d'Aubigné and Postel Criteria, 11 patients had very good results, and two had moderate results at the one-year follow-up. The average Merle d'Aubigné score was 14.6 on the final follow-up after one year.

Conclusion

Bipolar hemiarthroplasty in an unstable intertrochanteric femur fracture in the elderly results in better functional outcomes, as it helps in early full weight-bearing mobilization, which significantly decreases complications of prolonged immobilization and can be safely considered in the treatment of unstable intertrochanteric fractures in elderly patients.

## Introduction

Intertrochanteric femur fractures consist of roughly half of all hip fractures that occur due to low-energy mechanisms such as falling from a standing height. [1] These types of fractures are seen in specific populations with risk factors like elderly, female sex, history of trauma due to a fall, gait abnormalities, and osteoporosis. The major bone type in the intertrochanteric area is cancellous, extracapsular, and highly rich in blood supply, making it a good healing environment. Unstable intertrochanteric fractures are most commonly seen in patients with osteoporosis. These fractures are classified based on the stability and pattern of the break, with the Arbeitsgemeinschaft für Osteosynthesefragen/Orthopedic Trauma Association (AO/OTA) classification system being widely used. Type 31-A2 and A3 fractures typically denote unstable patterns, often involving comminution and a disrupted posteromedial cortex. The greater trochanter gives attachment to primary hip abductors, whereas lesser trochanters give attachment to primary hip flexors. The posteromedial bone’s thick strut, known as the calcar femorale, is important in the transfer of force from the neck to the shaft [2].

Restoring early mobility is the goal of treating any intertrochanteric fracture to limit the risk of medical problems from immobility and recumbency and return the patient to their preoperative state. When comparing results, the dynamic hip screw (DHS) was thought to be the gold standard, particularly for stable intertrochanteric fractures [3]. Since its introduction by the AO/Association of the Study of Internal Fixation (ASIF) group in 1998, the proximal femoral nail (PFN) has been a widely accepted treatment option for trochanteric fractures [4]. The PFN fixation has the benefit of decreasing the distance between the hip joint and the implant, which results in a more biomechanically stable construct [5].

Intertrochanteric fractures of the femur occur mainly in older adults. Some degree of osteoporosis is often seen in these patients, and the wide range of treatments available poses difficulty in choosing a specific treatment. As a sliding screw device encourages the impaction of the fracture, it was the implant of choice. When the posteromedial buttress is more comminuted than a simple lesser trochanteric fragment or when there are subtrochanteric fracture lines, the fracture is referred to as an unstable intertrochanteric fracture. In unstable fracture patterns, failure rates range from 8% to 25%, and in the most unstable fractures, they can reach 50% [6].

When compared with patients with femoral neck fractures, individuals with intertrochanteric fractures are slightly older and have a higher incidence of morbidity and mortality. Individuals in this age range typically have comorbidities or multiple systemic ailments, including diabetes and liver or cardiovascular disorders. Patients’ general conditions rapidly deteriorate as a result of these disorders, especially when bedridden. The objective of surgically treating these patients is to have them return to their previous activity status by permitting early complete weight-bearing mobilization [7]. For older patients with unstable intertrochanteric femur fractures, hemiarthroplasty with a bipolar prosthesis yields superior clinical outcomes in terms of early postoperative ambulation. The overall health and postoperative rehabilitation would be directly affected by this [8].

Keeping these facts in mind, the current study was undertaken to assess the functional outcomes of bipolar hemiarthroplasty in unstable intertrochanteric fractures in the elderly by using the Harris Hip Score (HHS) and Merle d'Aubigné and Postel Criteria [9,10].

## Materials and methods

Study design

The current prospective study was carried out at N. K. P. Salve Institute of Medical Sciences & Research Centre and Lata Mangeshkar Hospital, Nagpur, India, in the Department of Orthopedics from August 2016 to August 2018.

Ethical consideration

After discussing the study design with the board of the research committee and rectifying the ethical concerns that might originate from the course of the study, appropriate ethical approval from the institutional ethical committee, N. K. P. Salve Institute of Medical Sciences & Research Centre and Lata Mangeshkar Hospital was obtained, and then the study was conducted (approval number: 21/2016).

Study criteria and sample size estimation

After referring to similar articles and opinions from college statisticians, considering the feasibility and number of cases, a sample size of 15 was decided. A total of 15 elderly patients with unstable intertrochanteric femur fractures were included and received bipolar hemiarthroplasty treatment. Patients with unstable intertrochanteric fractures of the femur (AO classification 2.1 to 3.3) or osteoporosis (a Singh index of three or less) and those over 65 years of age were included in the study. The HHS and the Merle d'Aubigné and Postel Criteria were used to measure functional outcomes. Follow-ups of all the patients were done in the first, third, and sixth months after surgery and subsequently at the end of one year. Those patients who had stable intertrochanteric fractures were previously on ambulatory, were over age 65, had intertrochanteric fractures with neurological deficits, were unfit for anesthesia and surgery, and were not willing to undergo operative treatment were excluded from the study.

Assessment 

The assessment was discussed under the following headings: preoperative clinical assessment; radiology assessment; and postoperative assessment 

Preoperative clinical evaluation

Along with a systemic and general examination, the patient’s entire medical history was noted, including the mode of injury, related injuries, medical conditions, and state before the injury. The condition of the skin, surrounding soft tissue, and the fractured limb were also examined. Each patient had the standard hematological examinations. Skin traction was given to the injured limb on the first day following radiological studies. With excellent nursing care, the patient was prepared for surgery, and any potential difficulties from recumbency were minimized. Fitness for anesthesia was taken. The goal was to operate on the patient as soon as they were surgically fit, though the time between admission and operation varied.

Radiological evaluation

A standard anteroposterior view of the pelvis with both hip joints and a lateral view of the affected hip were taken in all cases to know the exact fracture pattern. The grade of osteoporosis was evaluated using the Singh index from the uninvolved side.

Procedure

Patients were operated on under spinal, epidural, or general anesthesia as deemed necessary by the anesthetist. Patients were taken in the left or right lateral position and secured using side clamps. The modified Hardinge approach was used in all cases. The femoral head was extracted using a head extractor. The transcondylar axis of the distal end of the femur was used as a guide for determining the anteversion of the prosthesis.

Determining the vertical and horizontal offsets in comminuted fractures to position the prosthesis properly proved to be challenging. We took advantage of trochanter anatomical landmarks. By utilizing a plastic ruler to match the femoral center of rotation horizontally with the greater trochanter’s tip, we were able to determine the approximate length of the abductor lever arm. The location of the shortened lesser trochanter was used to determine the anteversion. The greater trochanter was then fixed into position using stainless steel (SS) wires. The femoral canal was reamed to the proper diameter and stem size. Trial reductions with different neck lengths were used to measure limb length and evaluate the hip’s stability. After trials, the appropriate femoral stem was fixed using bone cement. The stability of the construct and restoration of limb length were assessed vigorously, and the wound was closed in layers over a negative suction drain.

Postoperative management

Active knee range of movement and full weight-bearing mobilization were initiated on the second day of surgery. The physiotherapist initiated straight leg raising, hip abduction, and quadriceps strengthening. On the 11^th^ postoperative day, the sutures were taken out. Patients were routinely monitored both clinically and radiologically following a predetermined procedure.

Assessment on follow-up

The 15 patients operated on for unstable intertrochanteric femur fractures with modular bipolar hemiarthroplasty were followed up postoperatively, clinically, and radiologically at one month, three months, six months, and one year. The functional outcome was evaluated using the HHS and the Merle d'Aubigné and Postel Criteria.

Statistical analysis

The data were analyzed statistically using analysis of variance (ANOVA). A p-value of <0.05 was regarded as significant.

## Results

Observations and results

The mean age of the patients was 78.73 ± 8.54 years, with an age range from 65 to 92 years. Most patients in the present study were women (11, 73%), and four (27%) were men. The maximum number of patients had fracture type A 2.3. According to AO classification, eight patients had fracture type A 2.3 (53%), four patients had type A 2.2 (27%), and three patients had type A 2.1 (20%).

Out of 15 patients, nine (65%) had grade 3 osteoporosis, and six (35%) had grade 2 osteoporosis, according to the Singh index. A total of eight patients (53.33%) had right-side fractures, and seven (46.67%) had left-side fractures.

In this current study, we found that five patients (33.33%) suffered from hypertension, three patients (20%) from diabetes mellitus, and two (13.33%) from hypothyroidism, whereas five (33.33%) had no comorbidities.

The blood loss in the current study averaged 350 ± 73.04 ml, with a range of 300 ml to 550 ml. The blood loss was calculated from blood collected in suction and mop counts, subtracting the volume of saline used in the wash. The average operative time was 119 ± 15.06 minutes, ranging from 105 to 150 minutes. The average number of days until mobilization was 3.4, ranging from two to 10 days. The mean length of stay was 12 days from the day of surgery, ranging from 11 to 15 days.

Two patients died after the first follow-up in the second and third month, respectively, due to reasons not related to surgery. No early or delayed complications were seen in any patients.

Average HHS and average Merle d'Aubigné score on follow-up

**Figure 1:**
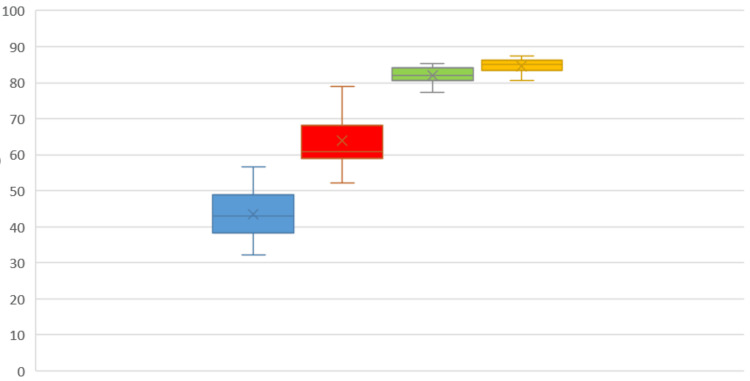
Box-whisker plots showing intragroup comparison of Harris Hip Score (HHS) recorded in sample population at first follow-up, second follow-up, third follow-up, and fourth follow-up, respectively; ANOVA F test value: 70.37, p <0.001 (highly significant) blue box: HHS at the one-month follow-up; red box: HHS at the three-month follow-up; green box: HHS at the six-month follow-up; yellow box: HHS at the one-year follow-up

**Figure 2:**
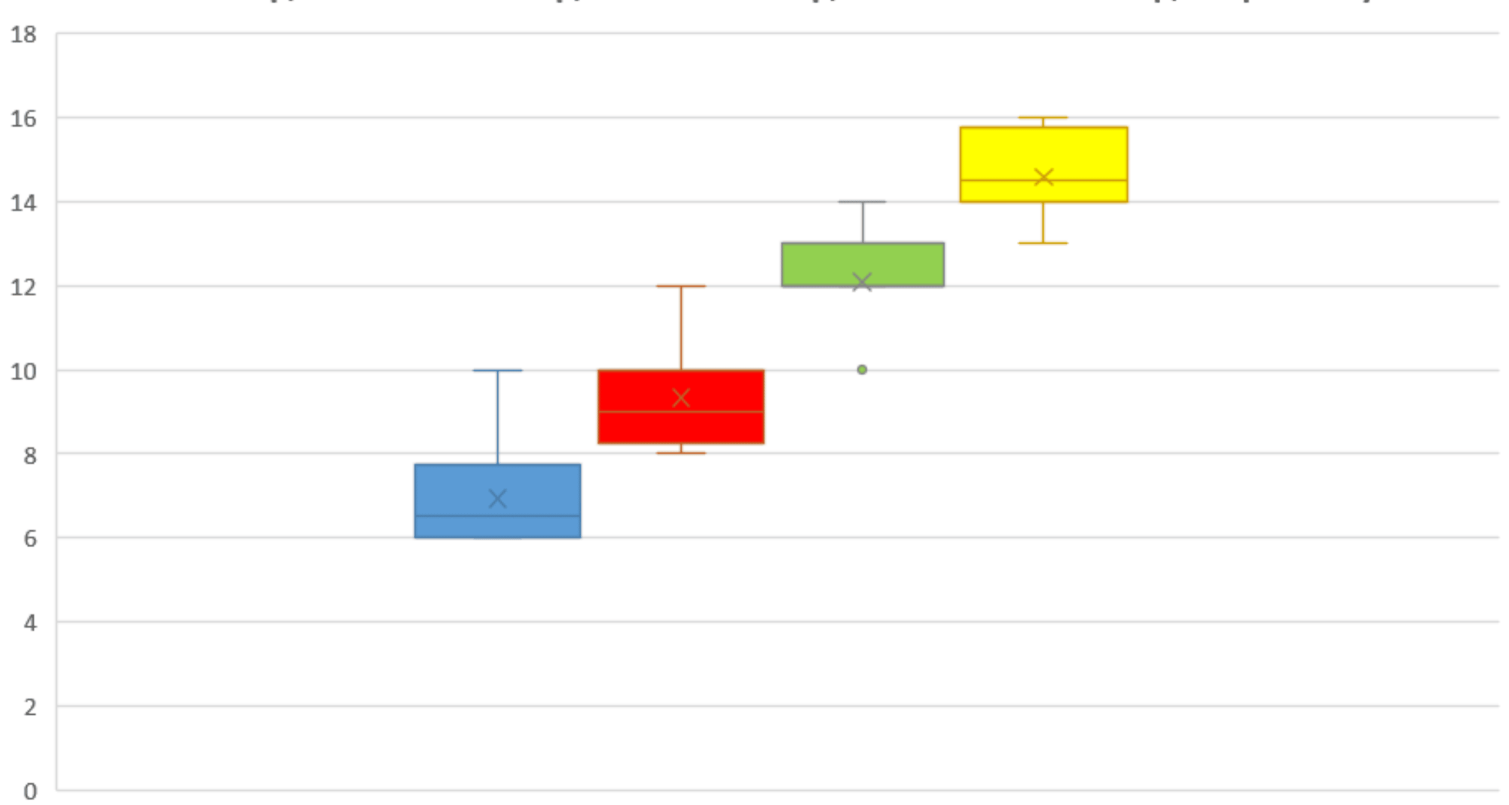
Intragroup comparison of Merle d'Aubigné score recorded in sample population at first follow-up, second follow-up, third follow-up, and fourth follow-up, respectively; ANOVA F test value: 63.72, p-value <0.001 (highly significant) blue box: Merle d'Aubigné score at the one-month follow-up; red box: Merle d'Aubigné score at the three-month follow-up; green box: Merle d'Aubigné score at the six-month follow-up; yellow box: Merle d'Aubigné score at the one-year follow-up

The mean HHS was 42.44 on the first follow-up, 64.43 on the second follow-up, 82.32 on the third follow-up, and 84.23 on the fourth follow-up. The average Merle d'Aubigné score was seven on the first follow-up, 9.46 on the second follow-up, 12.23 on the third follow-up, and 14.60 on the fourth follow-up. According to the ANOVA F test, the HHS in the interval between the first, second, third, and fourth follow-ups was calculated and found to be highly significant (p < 0.001). Similarly, the Merle d'Aubigné score was calculated in the interval between the first, second, third, and fourth follow-ups and was found to be highly significant (p < 0.001). Intragroup comparison of HHS and Merle d'Aubigné score was recorded at all the above-mentioned follow-ups, and it is depicted with a box-whisker plot (Figures 1-2).

## Discussion

With the view of reducing morbidity and mortality, operative treatment is recommended as the best option for intertrochanteric fractures in elderly individuals. Surgical techniques such as DHS and PFN are more reliable for managing stable intertrochanteric fractures and can safely initiate early mobilization. Conversely, long-term bedrest-related problems such as malunion, delayed union, and poor bone quality make managing an unstable intertrochanteric fracture more difficult.

For several decades, surgical procedures for intertrochanteric fractures in old individuals have been done through open/closed reduction and internal fixation by PFN, DHS, and, recently, proximal femoral nail antirotation (PFNA). Complications associated with DHS, PFN, and PFNA include the cutting out of the hip screw, the risk of instability, implant failure, mal reductions, infections, and occasionally the need for a second surgery, all of which can delay weight-bearing ambulation and early rehabilitation.

After fixation in these elderly patients, mobilization is delayed. Prolonged immobilization creates complications such as deep vein thrombosis, bed sores, pulmonary embolism, and pulmonary atelectasis supervenes. In the elderly age group, primary bipolar hemiarthroplasty done in cases of unstable intertrochanteric fractures facilitates early mobilization and thus may prevent complications of prolonged immobilization.

In the present study, the average age of patients was 78.73 years and found to be comparable with other studies done in the past, specifically, the mean age of 78.6 years by Sinno et al. [11] and 77.1 years by Sancheti et al. [12]. The vast majority of the patients in our study were women; 11 were women (73%), and four were men (27%). The average operative time in our study was found to be 119 minutes, which was less than the average operative time of 150 minutes reported by Vahl et al. [13].

In our study, the average amount of blood loss was 380 ml, which was comparable with the average blood loss of 350 ml reported by Sancheti et al. [12] and less than the average blood loss of 567 ml reported by Elmorsy et al. [14]. The average time taken for mobilization was 3.4 days in the present study, consistent with studies carried out by Sancheti et al. [12], with an average of 4.2 days, and Vahl et al., with an average of five days [13]. The average length of stay in the current study was 12 days, which was found to be less than in the study carried out by Harwin et al., where the average hospital stay was 15 days [15].

In our study group, 11 patients showed good results, and two showed fair results in one year, according to the HHS. Two patients died after the first follow-up due to causes not related to surgery. The average HHS was 84.23 on the final follow-up in our study group, which is consistent with the average HHS of 78.19 by Elmorsy et al. [14] and 80.35 by Sinno et al. [11].

In our study, 11 patients had good results, and two had moderate results, according to the Merle d'Aubigné and Postel Criteria at the one-year follow-up. The average Merle d'Aubigné score was 14.60 on the final follow-up after one year.

## Conclusions

The current study concluded that bipolar hemiarthroplasty, when performed by a skilled surgeon, can safely be regarded as an option for treating unstable intertrochanteric fractures in the elderly, especially those with advanced physiological age, osteoporosis, and comorbidities. This surgery can give predictable outcomes when performed correctly. This study indicated that bipolar hemiarthroplasty assisted elderly patients in early full weight-bearing mobilization and considerably reduced the problems of extended immobility, such as bed sores, deep vein thrombosis, pulmonary embolism, and atelectasis.
